# Comprehensive Genomic Analysis of *Meyerozyma guilliermondii* CECT13190: An Outstanding Biocontrol Agent

**DOI:** 10.3390/genes16020214

**Published:** 2025-02-12

**Authors:** Javier Vicente, José María Alonso de Robador, Beatriz Pintos, Arancha Gomez-Garay

**Affiliations:** Genetics, Physiology and Microbiology Department, Biology Faculty, Complutense University of Madrid, Ciudad Universitaria, S/N, 28040 Madrid, Spain; javievic@ucm.es (J.V.); jalonsod@ucm.es (J.M.A.d.R.); bpintos@ucm.es (B.P.)

**Keywords:** biocontrol agent, gene copy number variations (CNVs), secretome analysis, heat shock proteins (HSPs)

## Abstract

Background/Objectives: Biocontrol agents (BCAs) are gaining attention as sustainable alternatives to chemical pesticides. Understanding their molecular mechanisms is crucial for improving plant protection. This study investigates the genomic features of *Meyerozyma guilliermondii* CECT13190, a promising BCA, to identify key genes involved in its biocontrol abilities. Methods: Whole-genome sequencing of *M. guilliermondii* was performed, followed by bioinformatics analysis to identify genes and pathways related to biocontrol, including gene copy number variation (CNV) analysis. Gene ontology (GO) analysis was conducted to examine gene functions, and a comparative proteomics approach assessed the presence and role of proteins in the secretome of *M. guilliermondii*. Results: Genomic analysis revealed key biocontrol-related pathways. CNV analysis indicated a direct correlation between gene amplification and competitive fitness, with seven genes showing gains and five genes showing losses. GO analysis identified categories such as enzymes, transcription factors, ribosomal and proteasomal complexes, transporters, membrane proteins, RNA processing, and stress-response-related proteins. Secretome analysis identified *HSP70* and *HSP90* as potential effectors involved in biocontrol activity. Conclusions: This study provides insights into the genomic features of *M. guilliermondii* and its biocontrol potential. The identification of genes involved in the stress response and the secretome highlights the multifaceted mechanisms through which *M. guilliermondii* antagonizes plant pathogens. Practical outcomes include the identification of candidate genes and proteins, such as *HSP70* and *HSP90*, which can be targeted to enhance biocontrol efficiency in agricultural applications. Additionally, the observed CNVs offer a potential avenue for strain improvement programs to optimize competitiveness and efficacy in field conditions.

## 1. Introduction

Yeasts are invaluable in biotechnology and agriculture due to their remarkable versatility and environmentally friendly attributes. Historically, they have been extensively utilized in food and beverage fermentation, with *Saccharomyces cerevisiae* emerging as the most significant species in these processes [1]. In the realm of sustainable agriculture, yeasts play a dual role as biofertilizers and biopesticides. They promote plant growth through direct mechanisms such as nutrient solubilization and phytohormone production, as well as indirect mechanisms like biocontrol and soil bioremediation [2]. Moreover, yeasts effectively protect plants against pathogens by producing volatile organic compounds, killer toxins, and lytic enzymes [3]. Their rapid colonization of plant surfaces and ability to thrive under diverse environmental conditions underscore their potential as highly effective biocontrol agents. Beyond agricultural applications, yeasts also serve as exceptional model organisms in scientific research. Their eukaryotic origin and well-characterized genetic framework have significantly advanced our understanding of molecular biology and synthetic biology [4].

Among yeasts, *M. guilliermondii* has emerged as a promising agent for biocontrol and growth promotion across various crops. The yeast exhibits antagonistic mechanisms against plant pathogens, including the production of volatile organic compounds and hydrolytic enzymes [5]. *M. guilliermondii* has proven effective in managing fungal pathogens, particularly in postharvest fruits, and has been found to promote plant growth and photosynthesis in durum wheat, while also reducing the incidence of *Fusarium* crown rot [6]. Its efficacy against fungal pathogens such as *Fusarium oxysporum* and *Botrytis cinerea* is well-documented [6,7]. This yeast species enhances plant growth, improves photosynthetic efficiency, and activates defense mechanisms in crops including cucumber, tomato, and wheat [6,7]. Moreover, *M. guilliermondii* has been shown to mitigate abiotic stresses, such as heat and drought, further expanding its potential utility [7]. Specific plant responses include the promotion of early flowering in cucumber and significant increases in root and shoot lengths in tomato [6,7].

In addition to its agricultural impact, *M. guilliermondii* demonstrates significant biotechnological potential, particularly in enzyme production and the synthesis of bioactive metabolites for various industrial applications [8]. This non-conventional yeast exhibits unique properties that enhance its adaptability, such as its ability to metabolize a wide variety of carbon sources, including hydrophilic and hydrophobic materials, making it versatile for numerous biotechnological applications [8]. Additionally, *M. guilliermondii* contains a cold-adapted phenolic acid decarboxylase enzyme, enabling it to function effectively at low temperatures [9]. The yeast also demonstrates remarkable resilience to manganese stress by modulating its protein expression, particularly in genes related to DNA repair and oxidoreductase activity, highlighting its potential for use in bioremediation [10].

Recent advances in genomics have provided valuable insights into non-conventional yeasts. These species have garnered attention for their biotechnological potential and role in wine fermentation [8,11]. Furthermore, *M. guilliermondii* has shown promise in enzyme production and metabolite synthesis [8]. The availability of genomic sequences and annotations for these yeasts is expected to accelerate research and expand their industrial applications [11,12].

The primary objectives of this study are to assemble and analyze a high-quality genome of *M. guilliermondii* CECT13190, a strain with significant biocontrol and growth-promoting potential [13]. Additionally, the study seeks to investigate the genomic diversity of *M. guilliermondii* CECT13190 by comparing it to related strains, knowledge which will contribute to a deeper understanding of its biotechnological potential and applications.

## 2. Materials and Methods

### 2.1. Sequencing

DNA extraction was performed following the protocol described below. Yeast *M. guilliermondii* CECT13190 was cultured in potato dextrose broth (PDB) medium at 33 ± 2 °C for 72 h. Afterward, the culture was centrifuged at 10,000 rpm, and the supernatant was discarded. The pellet was resuspended in 300 μL of extraction buffer (200 mM Tris-HCl, pH 8.5, 250 mM NaCl, 25 mM EDTA, 0.5% SDS) and 150 μL of 3 M sodium acetate (pH 5.2), then incubated at −20 °C for 10 min. Following incubation, the mixture was centrifuged at 10,000 rpm for 5 min, and the supernatant was transferred to a new tube, to which an equal volume of isopropanol was added. The sample was incubated at room temperature for 5 min, followed by centrifugation at 10,000 rpm for 5 min. The resulting pellet was washed with 70% ethanol, air-dried, and resuspended in 50 μL of TE buffer (Tris-EDTA). DNA was stored at −20 °C until further use.

DNA quantification was performed using two methods: the NanoDrop One (Thermo Scientific, Waltham, MA, USA) spectrophotometer, which relies on ultraviolet absorbance for quantifying DNA, and the Qubit 4 Fluorometer (Thermo Scientific), which utilizes fluorescence-based DNA quantification. Both methods enable accurate determination of nucleic acid concentration, which is crucial for library preparation and ensuring sequencing accuracy. DNA purity was assessed by evaluating the A260/280 and A260/230 absorbance ratios. An A260/280 ratio of approximately 1.8 indicates pure DNA, and deviations from this value suggest potential protein contamination. Additionally, the A260/230 ratio was used as a secondary measure of purity, indicating the presence of chemical contaminants remaining from the DNA isolation process.

Genomic sequencing was conducted using the Illumina MiSeq platform at the CAI (Centro de Apoyo a la Investigación) de Técnicas Biológicas (Unidad de Genómica) at the Universidad Complutense de Madrid (UCM). The sequencing yielded paired-end reads (2 × 150 bp), providing a comprehensive genome coverage with high resolution.

Quality control of the sequencing data was performed using FastQC [14], a bioinformatics tool that assesses various quality parameters, including base quality distribution, the presence of adapter sequences, and duplication levels. After quality evaluation, sequences were cleaned to remove low-quality reads using Seqtk (https://github.com/lh3/seqtk, accessed on 15 February 2024), applying a filter of Q > 28, ensuring that only high-quality sequences were retained for downstream analysis.

### 2.2. Variant Calling

Variant calling is a bioinformatics process used to identify genetic differences (variants) between the sequenced sample and a reference genome. These variants include single nucleotide polymorphisms (SNPs), insertions, deletions, and duplications. The variant calling process involves aligning the sequencing reads to a reference genome and comparing the sequences to identify positions with differences, which are considered potential genetic variants.

The process involves two main steps:

1. Reference strain indexing (ATCC 6260, *M. guilliermondii*): the reference strain was indexed using BWA (Burrows–Wheeler aligner, v0.7.17-r1188) [15], allowing the mapping of sequencing reads to the reference genome.
2. SAM and BAM file generation: SAM (sequence alignment/map) files were generated and converted to the BAM (binary alignment/map) format using samtools (v1.6). These files contain the aligned sequencing reads and serve as the basis for variant identification.

### 2.3. Segmental Duplications and Gene Copy Number Variant Analysis

Segmental duplications are genomic regions resulting from duplication events. Analyzing the coverage of these regions involves comparing the proportion of reads aligned to duplicated versus non-duplicated regions. To identify segmental duplications across the genome, 1 kb windows were analyzed using BAM files and processed using GenomicRanges, GenomicAlignments, and Parallel R packages (v 4.4.0).

To determine the copy number variants (CNVs) of the genes present in the reference strain, we analyzed the *M. guilliermondii* CECT13190 BAM file using control-FREEC (v11.4) (window = 1; breakPointThreshold = 0.05; minExpectedGC = 0.35; maxExpectedGC = 0.55; ploidy = 1).

### 2.4. Genome Assembly and Supplemental Gene Determination

Genome assembly was conducted using SPAdes Genome Assembler (v3.13.0) [16], which applies multiple algorithms to assemble sequencing reads into contiguous sequences (contigs). The assembly process tested various k-mer sizes (odd values from 63 to 127), as the optimal k-mer size impacts assembly quality and contiguity. The final k-mer size of 101 was selected based on the total number of contigs and the N50 value. The goal was to choose the k-mer size that produced an assembly with fewer, larger contigs, indicating a more complete representation of the genome. To determine the content of supplemental genes in the *M. guilliermondii* CECT13190 genome compared to the reference strain, a previously described methodology was employed [17]. Briefly, Proteinortho (v6.0.18) [18] was used to perform all-against-all sequence similarity searches using BLASTp. Predicted protein sequences were generated by Augustus, utilizing training sets based on the *M. guilliermondii* reference proteome. Genes present in the newly sequenced strain and not in the reference one were considered as additional ones and further annotated.

### 2.5. Phylogenetic Analysis

Several species, including *M. guilliermondii*, *M. carpophila*, *M. caribbica*, *M. athensensis*, and *Debaryomyces hansenii* (used as an outgroup), were selected for phylogenetic analysis. Conserved gene sequences, including 28S rRNA, actin, ITS, RPB2 (RNA polymerase subunit II), TEF (translation elongation factor), and tubulin, were analyzed using BLAST^®^ (basic local alignment search tool) [19] to compare gene sequences between species. All homologs were aligned with MAFFT (v7.520) using default parameter values [20] and further concatenated. A maximum likelihood tree was constructed using 100 iterations and visualized using the ggtree R package (v3.8.0). Pairwise distance (cophenetic) between strains was calculated using the ape R package (v5.8).

### 2.6. Gene Annotation

Protein prediction was performed using genome and proteome data from the reference strain. Genes without orthologs in the reference strain were considered supplementary and retained for further analysis. Gene annotation was performed using two databases: Koala (KEGG Orthology and Links Annotation) [21] and EggNOG (v2.1.12) [22], providing functional insights into the identified genes. In both cases, only significant annotations (e-value < 1 × 10^−4^) were maintained for further consideration.

### 2.7. Functional Analysis

Gene ontology (GO) analysis was carried out using DAVID Bioinformatics Resources (Database for Annotation, Visualization and Integrated Discovery), which offers tools for the functional annotation of genes. The online DAVID interface was used to upload gene sets, performing functional analysis to determine biological processes, cellular components, and molecular functions associated with the identified genes using default parameters. Additionally, KEGG (Kyoto Encyclopedia of Genes and Genomes) pathways were analyzed to identify associated biological pathways, retaining only significant annotations (e-value < 1 × 10^−4^).

### 2.8. Plant Material and Treatments

For the biocontrol experiments conducted with *Vitis vinifera* L., a total of 18 glass jars (17 × 25.5 × 7 cm) were utilized. These jars were thoroughly cleaned using soap, bleach, and distilled water before being filled with sterilized vermiculite. The filled jars were then autoclaved at 121 °C for 20 min to ensure sterilization.

The plant material used consisted of 18 grafted grapevine plants of the certified *Tempranillo* clone RJ51/110R-E35 which had been previously rooted. To disinfect the plants, they were first submerged in 70% ethanol for 5 min, followed by a 10 min treatment with a 4% bleach solution. Afterward, the plants were rinsed three times in autoclaved distilled water, with each rinse lasting 5 min.

The plants were placed in the prepared glass jars, ensuring that the roots were well covered by the vermiculite. The experiment was conducted under controlled environmental conditions, with a temperature maintained at 20 ± 2 °C and a photoperiod of 16 h of light followed by 8 h of darkness. The plants were irrigated with filtered water to maintain appropriate moisture levels in the vermiculite. Additionally, once a week, they received a 1:10 dilution of Hoagland No. 2 nutrient solution, containing macronutrients such as nitrogen (N) at 14 mM, phosphorus (P) at 1 mM, potassium (K) at 5 mM, calcium (Ca) at 4 mM, magnesium (Mg) at 2 mM, and sulfur (S) at 2 mM. Micronutrients included iron (Fe) at 20 μM, manganese (Mn) at 2 μM, zinc (Zn) at 2 μM, copper (Cu) at 0.5 μM, molybdenum (Mo) at 0.1 μM, and boron (B) at 25 μM. Phenological observations were recorded following bud break.

Each plant’s phenological status was monitored throughout the course of the experiment using the phenological scale proposed by Baggiolini [23]. The experimental trials involving yeast inoculation were initiated when the vines had fully developed leaves and tendrils, corresponding to phenological stage E (extended leaves). Each treatment group consisted of three biological replicates, with each replicate representing a pool of three plants. This resulted in a total of nine plants per treatment, distributed across three biological replicates. The entire experiment was repeated twice, allowing for two independent repetitions of the study.

The two treatment groups for the inoculation experiment were as follows:

- Group control: the vines were watered with filtered tap water only.
- Yeast inoculated vines group: the vines were inoculated with a yeast suspension, prepared by growing the yeast in PDB medium for 48 h at 120 rpm agitation. The final suspension contained approximately 8 × 10^8^ CFU/mL.

Each plant was treated with a precise volume of inoculum, included in the irrigation water, in its respective pot. A total of 25 mL was applied to each plant, ensuring a consistent distribution and adequate contact with the root system.

### 2.9. Proteomic Analysis

For the proteomic analysis, each biological replicate consisted of a pool of three plants, with three biological replicates per treatment. Samples were precipitated using TCA/acetone and analyzed in duplicates. The resulting pellets were resuspended in 40 μL of 100 mM TEAB (triethylammonium bicarbonate) buffer. Protein quantification was performed using fluorometric detection on a Qubit fluorometer (Thermo Fisher Scientific, Waltham, MA, USA).

Samples underwent reduction with 10 mM DTT at 37 °C for 60 min, followed by alkylation with 25 mM iodoacetamide in the dark for 60 min. Afterward, 0.25 μg of recombinant sequencing-grade trypsin (Roche Molecular Biochemicals, Graz, Austria) in 25 mM ammonium bicarbonate buffer (pH 8.5) was added, and digestion proceeded overnight at 37 °C. Peptides generated from digestion were concentrated by vacuum centrifugation (SpeedVac, Thermo Scientific, Bremen, Germany), reconstituted in 12 μL of 2% acetonitrile (ACN) with 0.1% formic acid (FA), and stored at −20 °C until analysis by LC-MS/MS.

Peptide separation was performed using nano-liquid chromatography (nano Easy-nLC 1000, Thermo Scientific, MA, USA) coupled with a Q-Exactive HF high-resolution mass spectrometer (Thermo Scientific, Bremen, Germany). A pre-column (Acclaim PepMap 100, Thermo Scientific, Bremen, Germany; 20 mm × 75 μm ID, 3 μm particle size, 100 Å pore size) was used for online concentration, followed by separation on a C18 Picofrit column (Thermo Scientific Easy Spray Column, PepMap RSLC C18n, Bremen, Germany; 500 mm × 75 μm ID, 2 μm particle size, 100 Å pore size) at a flow rate of 250 nL/min. Peptides were eluted using a gradient of buffer B (ACN with 0.1% FA) from 2% to 35% over 150 min, followed by a ramp from 35% to 45% over 10 min (buffer A: 0.1% FA in water). The mass spectrometer was equipped with an Easy nanoelectrospray source for ionization. The ion transfer tube temperature was set to 290 °C, and data acquisition was carried out in a data-dependent acquisition (DDA) mode, where the full scan MS mode was performed with a resolution of 60,000 over a mass range of m/z 350–1800 Da. MS/MS data were acquired with a resolution of 30,000. Up to 15 precursor ions with charge states between 2+ and 4+ were selected per microscan based on intensity (threshold: 1 × 10^4^), with a dynamic exclusion window of 10 s. The selected precursors were isolated with a window width of ±2 m/z units and fragmented by high collision dissociation (HCD) at a normalized collision energy of 20%.

The acquired data were analyzed using Proteome Discoverer 2.4 (Thermo Scientific) with the Mascot 2.6 search engine (MatrixScience.com). The following protein databases were searched:

- UP-*V. vinifera* (160,461 sequences), downloaded from UniProt (https://www.uniprot.org).
- UP-*M. guilliermondii* (5974 sequences), downloaded from UniProt.

Search parameters included a peptide precursor tolerance of 10 ppm, a fragment tolerance of 0.02 Da, with up to two missed cleavages allowed for trypsin digestion. Carbamidomethylation of cysteine was set as a fixed modification, while oxidation of methionine and N-terminal acetylation were considered variable modifications. Proteins were considered “correctly” identified if they met the following criteria: a false discovery rate (FDR) of less than 1% and at least one uniquely identified peptide with a confidence interval (CI) greater than 99%. This ensures that the probability of a false-positive identification is less than 1% (q-value < 0.01).

### 2.10. Effector Prediction in the Secretome

EffectorP [24], a machine-learning-based bioinformatics tool, was used to predict the ability of proteins to function as effectors in plants. Protein sequences detected in the proteomic analysis of *M. guilliermondii*-inoculated grapevine plants were analyzed with EffectorP to assess their likelihood of acting as effectors in the plant cytoplasm or apoplastic space using default parameters.

## 3. Results

### 3.1. Sequencing

The DNA concentration was 30.45 ng/μL, and its purity was assessed as suitable for sequencing through the 260/280 nm absorbance ratio. This ratio confirmed that the DNA was free of protein contaminants, with a measured value of 1.938, indicating adequate purity for high-quality sequencing.

Illumina sequencing generated 10,514,805 high-quality reads, with an average quality score above 32. A quality filter was applied to retain only the reads with a quality score (Q) higher than 28. This threshold was selected based on standard recommendations to ensure data reliability, as a quality score above 28 guarantees a low error rate in the reads and improves the accuracy of the genomic assembly.

### 3.2. Assembly

The genomic characteristics of the sample are summarized in Table 1. A K-mer value of 101 was selected, resulting in a low number of contigs and a high N50 value. This K-mer value was chosen based on a preliminary analysis of the contig size distribution and recommendations from the literature for this type of sequencing. The K value was selected to optimize the generation of a small number of contigs, ensuring better continuity in the assembly, and to achieve a high N50 value, indicating a good representation of the genome with long contigs.

The genome assembly of *M. guilliermondii* CECT13190 displays promising characteristics. With an N50 value of 1,674,950 and contig lengths ranging from a minimum of 7954 to a maximum of 2,085,238, this assembly suggests a solid and diverse representation of the genome. The N50 value of 1,674,950 indicates that half of the genome is represented by contigs of at least that length, demonstrating good continuity in the assembled genomic sequence. These results suggest a high-quality assembly, providing a solid foundation for further analysis.

The read count serves as a measure of the abundance or frequency of each genetic variant in the sample. Variants with a high read count are generally considered more reliable, as they are supported by a greater amount of sequencing evidence. Given the genome assembly of *M. guilliermondii* CECT13190, a read count of 2000 would be considered adequate for genetic variant analysis in this context. This value provides sufficient coverage to support variant calls across a broad range of genomic regions, ensuring reasonable confidence in the results.

The read counts represent the number of times each variant is observed in the sequencing dataset. During DNA sequencing, the process generates numerous short reads that represent fragments of the genome. Each read has an associated quality score that indicates its reliability. In variant calling, differences between the sample genome (*M. guilliermondii* CECT13190) and the reference genome (*M. guilliermondii* ATCC 6260) are identified, including SNPs (single nucleotide polymorphisms), insertions, or deletions.

A total of seven genes with copy gain and five genes with copy loss were identified in the CECT13190 strain compared to the ATCC 6260 strain (Table 2). These genomic variations suggest the presence of gene duplication or deletion events in the *M. guilliermondii* CECT13190 strain which could have significant implications for its biology, phenotype, and adaptation to different environmental conditions. These findings highlight the genomic diversity within *M. guilliermondii* and underscore the importance of detailed studies to better understand genetic variability and its impact on yeast biology.

### 3.3. Phylogenetic Analysis

**Figure 1:**
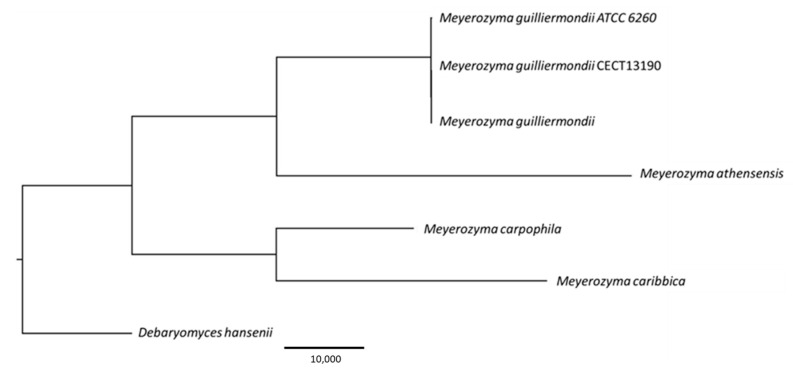
Phylogenetic relationships between the *M. guilliermondii* CECT13190 strain and other related species, including *M. carpophila*, *M. caribbica*, *M. athensensis*, and *D. hansenii*. It also compares the strain with an additional *M. guilliermondii* isolate from *Cannabis sativa* and the reference strain ATCC 6260. A maximum likelihood tree was constructed using 100 iterations.

The results from the phylogenetic analysis, based on six specific markers (28S, actin, ITS, RPB2, TEF, and tubulin), confirmed the taxonomic assignment of the studied isolate. Additionally, it was observed that the *M. guilliermondii* CECT13190 strain is more closely related to another *M. guilliermondii* strain isolated from *Cannabis sativa* (divergence = 0.080%) than to the reference strain (ATCC 6260) used in the study (divergence = 0.125%) (Figure 1).

### 3.4. Bioinformatic Analysis

A total of 354 genes were identified as supplementary in *M. guilliermondii* CECT13190, representing approximately 7% of the total genes analyzed. Using two distinct databases, Koala and EggNog, a total of 170 of these genes were annotated. Of these 170 annotations, 120 were found to be exclusive to this strain and absent in the closest strain based on the phylogenetic analysis. This result indicates an important differentiation not only in terms of genomic similarity but in gene content.

### 3.5. Functional Analysis

**Figure 2:**
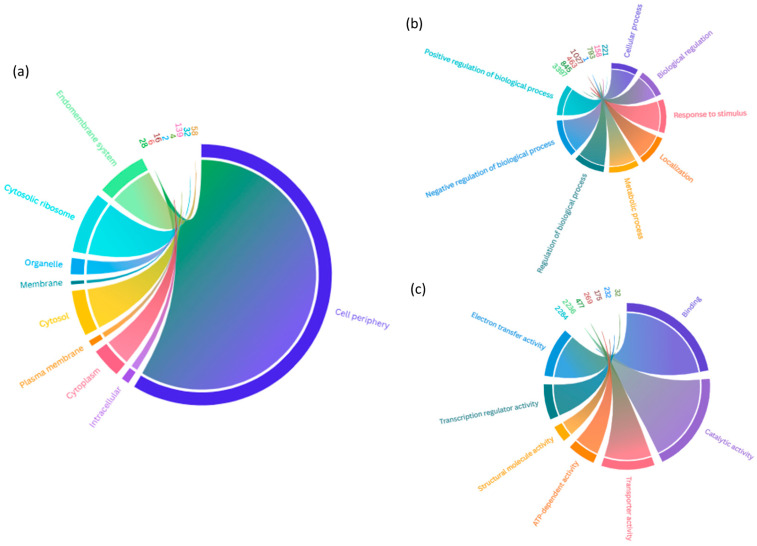
Gene ontology (GO) analysis of differential proteins in *M. guilliermondii* CECT13190. (**a**) Cellular component; (**b**) biological process; (**c**) molecular function. Number of proteins in each GO term is indicated.

Gene ontology (GO) analysis highlighted categories such as enzymes and catalytic activities, transcription factors and gene expression regulators, components of ribosomal and proteasomal complexes, transporters and ion channels, membrane proteins and organelles, proteins involved in organelle biogenesis, RNA processing factors, and nuclear transport proteins, as well as stress-response-related proteins (Figure 2 and Figure S1).

The proteins identified exclusively in the *M. guilliermondii* CECT13190 yeast may have specific functions in plant–microbe interactions. While many of the proteins may not be directly relevant to the defense response, others could play a critical role in the interaction between the yeast and the plant.

### 3.6. Proteomic Analysis

In the proteomic analysis of grapevine plants inoculated with *M. guilliermondii* CECT13190, HSP70 and HSP90 proteins from the yeast were identified, suggesting their presence in the secretome of this strain. Since both heat shock proteins could function as effectors in plant defenses, an analysis using the EffectorP program was performed. The results revealed that HSP90 has a 0.802 probability of being a cytoplasmic effector and a 0.753 probability of being an apoplastic effector, while HSP70 has a 0.902 probability of acting as a cytoplasmic effector.

## 4. Discussion

Genomic analysis of biocontrol agents (BCAs) is essential to understand their mechanisms of action and enhance their efficacy against plant pathogens. Advances in omics approaches have previously unveiled the molecular basis of BCA activity [25]. Whole-genome sequencing has been employed in identifying genetic features such as secondary metabolite biosynthesis clusters, CAZymes, and unique genes critical for antimicrobial properties [26]. For example, the genome of *Pseudomonas fluorescens* Pf-5 has provided insights into its rhizosphere survival strategies and broad-spectrum antibiotic production [27]. These genomic resources are essential for optimizing BCA performance, boosting production, and assessing non-target effects, contributing to sustainable and eco-friendly agricultural practices [28]. The genomes of biocontrol yeasts, such as *Papiliotrema terrestris* LS28 (21.29 Mb), *Rhodotorula kratochvilovae* LS11 (22.56 Mb), and *Candida oleophila* I-182 (14.73 Mb), have been sequenced and annotated, revealing numerous protein-encoding genes with multiple biotechnological roles [29,30,31]. Comparative analysis of *C. oleophila* has highlighted unique protein families linked to biocontrol efficacy [31]. These yeasts demonstrate antagonistic activity against various plant pathogens in field and postharvest settings. *Hanseniaspora opuntiae* releases compounds with local and systemic protective effects against necrotrophic fungi. Transcriptomic analysis of *Arabidopsis thaliana* treated with these compounds has provided valuable insights for developing eco-friendly alternatives to synthetic fungicides [32].

*M. guilliermondii* is notable for its biotechnological applications, biocontrol potential, and clinical relevance [33]. The genome of strain vka1, isolated from organic compost, contains 5385 genes related to the composting potential [34]. Conversely, the genome of the strain SO, from spoiled orange, has revealed virulence factors such as SAP, PLC, and PLD genes, highlighting potential clinical concerns [35]. The availability of the complete genome for strain ATCC 6260 and genetic tools has enabled research into metabolic engineering and pathogenicity [11]. Comparative genomics has identified gene copy number variations (CNVs) in *M. guilliermondii* CECT13190 versus ATCC 6260, uncovering eight genes with copy gains (e.g., cytochrome B, urea carboxylase) and five with losses, although no statistical analysis could be performed as only one strain was analyzed. CNVs contribute to genomic plasticity, enabling adaptation to environmental conditions [36,37]. For instance, cytochrome B copy gain enhances respiratory efficiency, supporting defense mechanisms and antifungal compound production [38]. The ability of *M. guilliermondii* CECT13190 to fix atmospheric nitrogen and utilize various nitrogen sources like urea and amino acids confers adaptability and competitiveness in nitrogen-limited environments. Key genes, such as urea carboxylase and amino acid permease, facilitate these processes. Nitrogen assimilation strategies enhance growth and biocontrol efficacy by improving competitiveness in agricultural ecosystems [39,40,41].

The gain of a bHLH domain protein gene enhances the defense coordination of *M. guilliermondii*, regulating antimicrobial compound production in response to environmental stimuli [42]. Additionally, the yeast’s capacity to oxidize alcohols into aldehydes and hydrogen peroxide improves adaptability and contributes to pathogen defense. The gain of OPT family transporter genes improves nutrient uptake efficiency in resource-limited environments, optimizing growth and persistence [43,44,45]. Syntaxins, essential for vesicular transport and membrane fusion, also play crucial roles. Homologs in yeasts, such as Sso1p and Sso2p, facilitate secretory vesicle fusion with the plasma membrane, underscoring their importance in intracellular trafficking [46,47].

The absence of certain genes in *M. guilliermondii* CECT13190, such as glutamate synthase, may be compensated by the activity of glutamine synthetase. This enzyme could synthesize glutamine, which can then be converted into glutamate. Insights from *S. cerevisiae* show that its NADH-dependent GOGAT enzyme, encoded by the *GLT1* gene, consists of three identical subunits of 199 kDa and plays a secondary role in glutamate biosynthesis [48]. Instead, the primary pathway relies on NADP–glutamate dehydrogenase [49]. *S. cerevisiae* also efficiently utilizes organic nitrogen sources like amino acids and peptides through specialized transporters, including those of the OPT family.

The ABHD protein family, known for its α/β hydrolase domain, has critical roles in lipid metabolism and various physiological processes. For example, ABHD11 in *Arabidopsis* functions as a lysophospholipase, with its alteration leading to increased polar lipid accumulation and enhanced growth [50]. These genomic variations in *M. guilliermondii* CECT13190 highlight its genetic flexibility, enabling it to adapt to environmental changes, resist stressors, and potentially exhibit modified pathogenicity. Further functional studies could provide valuable insights into how these adaptations contribute to the strain’s biocontrol properties and agricultural potential.

Enrichment in stress-related GO categories suggests that the strain is equipped to withstand osmotic, oxidative, and thermal stresses, commonly encountered in agricultural settings. Stress-responsive genes help maintain cellular integrity through the production of protective proteins (e.g., chaperones, antioxidants) and metabolic adjustments under adverse conditions. Studies have shown that yeast stress-responsive genes exhibit distinct features, with transcription factor targeting and gene conservation being highly predictive of their roles [51]. Stress-induced expression is often enriched in evolutionarily young genes, indicating a shared adaptation mechanism among budding yeasts [52,53]. This capability likely allows *M. guilliermondii* to thrive in challenging environments, enhancing its effectiveness as a biocontrol agent. The enrichment of protein-synthesis-related GO categories further underscores the strain’s adaptability. Efficient protein synthesis is critical for rapid environmental adaptation, ensuring proper growth, maintenance, and stress response [54]. Transport-related GO categories emphasize the strain’s adaptability in nutrient-limited environments, such as the rhizosphere. Transporters involved in nitrogen uptake (e.g., urea, amino acids) provide a competitive advantage in nitrogen-poor soils. This nutrient acquisition flexibility is crucial for fungal–plant interactions, as fungi exploit host resources and influence plant metabolism. Plants selectively recruit beneficial microbes through root exudates, which improve their nutrition and stress tolerance [55,56,57]. Additionally, transporters related to the secretion of antimicrobial compounds and cell-wall-modifying enzymes enable *M. guilliermondii* to suppress pathogenic microorganisms, further enhancing its biocontrol potential [58,59,60]. This capability may enable *M. guilliermondii* CECT13190 to respond effectively to environmental changes and maintain cellular function under stress.

Proteomic analysis of grapevine plants inoculated with *M. guilliermondii* CECT13190 identified heat shock proteins (*HSP*) 70 and 90 in the yeast’s secretome, suggesting their potential involvement in plant–host interactions. HSPs, typically associated with protein folding and stabilization under stress, may function as effectors in plant defense mechanisms in this context. Both *HSP70* and *HSP90* play pivotal roles in plant immunity, contributing to the hypersensitive response and non-host resistance [61]. *HSP90* activates plant defenses and can trigger immune responses in mammals, highlighting the conservation of these pathways across species [62]. *HSP70* enhances effector-induced cell death while suppressing pathogen growth, emphasizing its importance in basal defense [63]. Elicitors, such as those found in *M. guilliermondii*, stimulate plant innate immunity by activating signalling cascades through plant receptors [64,65]. These findings suggest significant biotechnological potential for *M. guilliermondii* CECT13190 in viticulture. Effectors, which modulate host immunity, can operate in apoplastic or cytoplasmic spaces [66,67] and exhibit dual roles of suppression or activation [68]. The analysis of *HSP90* and *HSP70* in *M. guilliermondii* CECT13190 revealed high probabilities for both cytoplasmic and apoplastic effector functions. Cerato-platanins, small, secreted proteins, exemplify this duality, acting either as virulence factors or as defense inducers [69]. In plants, HSPs help maintain protein homeostasis and regulate resistance proteins under stress [70]. In animals, intracellular HSPs provide cytoprotection, while extracellular HSPs elicit immune responses [71]. The ability of *M. guilliermondii* CECT13190 to regulate defensive proteins and genes in *V. vinifera* highlights its potential for enhancing grapevine pathogen resistance [72]. The strain role as a biocontrol agent offers promise as a sustainable alternative to pesticides. However, translating laboratory findings to field applications remains a challenge. Efforts are focused on improving treatment efficacy in uncontrolled environments and advancing sustainable crop protection strategies.

The use of biocontrol agents, like *M. guilliermondii* CECT13190, as an alternative to pesticides in crop protection holds significant promise. However, translating findings from controlled conditions to field applications remains a major challenge. Current research focuses on enhancing the efficacy of treatments in non-controlled environments and advancing sustainable strategies for crop protection.

## 5. Conclusions

In conclusion, the genomic and functional versatility of *M. guilliermondii* CECT13190 positions it as a promising biocontrol agent with significant potential in sustainable agriculture. Genomic analysis has provided valuable insights into its adaptability, biocontrol potential, and resilience in various environments. The identification of key gene copy gains and losses, along with enrichment in stress-response and transport-related gene ontology categories, underscores the yeast’s capacity to survive and thrive under challenging conditions. Its ability to efficiently synthesize proteins, secrete bioactive compounds, and produce antimicrobial metabolites highlights the strain’s suitability for combating plant pathogens and promoting plant health. Additionally, nitrogen utilization and the ability to fix atmospheric nitrogen enhance its competitiveness in agricultural ecosystems, enabling an effective interaction with plant hosts. The presence of genes involved in plant defense mechanisms, such as secretome HSPs, further reinforces its role as an eco-friendly biocontrol agent.

These traits, combined with its capacity to adapt to fluctuating environments, position *M. guilliermondii* CECT13190 as a promising alternative to chemical pesticides, contributing to ecological sustainability. As advances in genomic analysis continue, they offer critical insights into optimizing the application of this strain in agricultural practices, ultimately enhancing crop protection and promoting sustainable farming methods. Future research focusing on the functional characterization of these genomic features will be crucial for improving the efficacy and broader application of *M. guilliermondii* CECT13190 in sustainable agricultural practices.

## Figures and Tables

**Table 1:** Genome characteristics of the *M. guilliermondii* CECT13190 strain.

Genome Characteristics	Value
Contigs	142
Contigs (≥1000 bp)	15
Number of contigs greater than N50	3
Maximum contig length	2,085,238
Minimum contig length	7954
Genome size	10.64 Mb
N50	1,674,950
No. of genes	5057

**Table 2:** Gene copy gains and losses in the *M. guilliermondii* strain CECT13190 compared to the reference strain ATCC 6260.

Gene Copy Gains		
Gene Name	Function	Copies
Hypothetical protein PGUG_05919	Cytochrome B	16
Hypothetical protein PGUG_04496	Urea carboxylase	15
Hypothetical protein PGUG_04290	Amino acid permease/SLC12A domain-containing protein	2
Hypothetical protein PGUG_04425	BHLH domain-containing protein	2
Hypothetical protein PGUG_03467	Long chain alcohol oxidase	2
Hypothetical protein PGUG_03612	OPT family small oligopeptide transporter	2
Hypothetical protein PGUG_04423	Sintaxin N-terminal domain-containing protein	2
Gene copy losses		
Conserved hypothetical protein	Glutamate synthase (NADH)	0
Hypothetical protein PGUG_02897	AB-hydrolase-1 domain-containing protein	0
Hypothetical protein PGUG_03613	GSKIP domain-containing protein	0
Hypothetical protein PGUG_03126	Major facilitator superfamily (MFS) profile domain-containing protein	0
Hypothetical protein PGUG_03125	NADPH-dependent 1-acyldihydroxyacetone phosphate reductase	0

## Data Availability

The data presented in this study can be accessed on NCBI Bioproject: PRJNA1211854. Other datasets are available on request from the authors.

## Supplementary Materials

The following supporting information can be downloaded at: https://www.mdpi.com/article/10.3390/genes16020214/s1, Figure S1: Heatmap of Gene Ontology (GO) enrichment analysis. The heatmap includes the diversity and relevance of the enriched GO terms across functional categories. Rows represent significantly enriched GO terms, while columns represent the different biological processes associated with them.
