# Supplementary material for: Comprehensive Genomic Analysis of Meyerozyma guilliermondii CECT13190: An Outstanding Biocontrol Agent
Source: Genes (Basel). 2025 Feb 12;16(2):214. doi: 10.3390/genes16020214 (PMC11855519; doi:10.3390/genes16020214)
Supplement: Supplementary file 1 [file genes-16-00214-s001.zip › genes-3423425-supplementary.pdf]

Supplementary Material

Comprehensive Genomic Analysis of *Meyerozyma guilliermondii* CECT13190: An Outstanding Biocontrol Agent

Javier Vicente, José María Alonso de Robador, Beatriz Pintos and Arancha Gomez-Garay *

Genetics, Physiology and Microbiology Department, Biology Faculty, Complutense University of Madrid, Ciudad Universitaria, S/N, 28040 Madrid, Spain; javievic@ucm.es (J.V.); jalonsod@ucm.es (J.M.A.d.R.); bpintos@ucm.es (B.P.)

*Correspondence: magom02@ucm.es

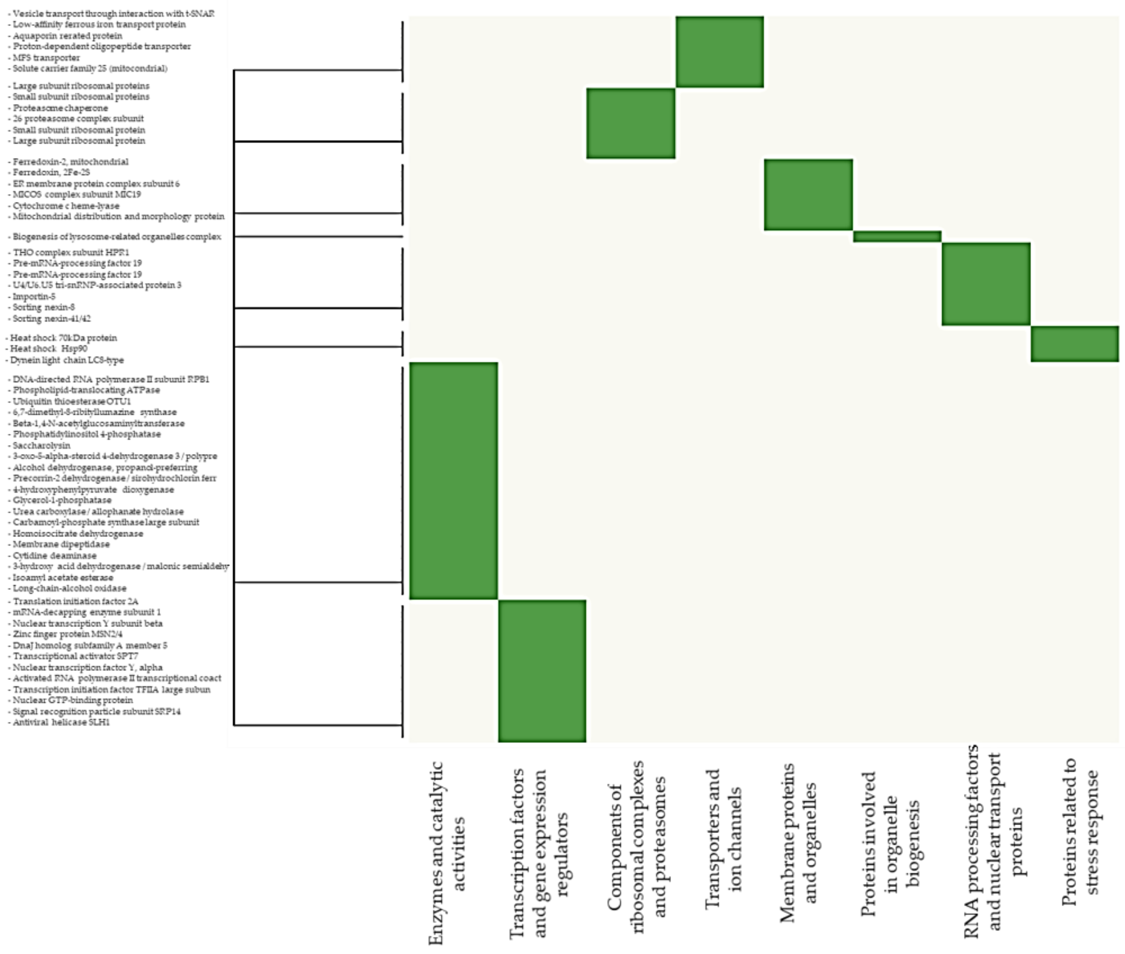

Supplementary Figure S1. Heatmap of Gene Ontology (GO) enrichment analysis. The heatmap includes the diversity and relevance of the enriched GO terms across functional categories. Rows represent significantly enriched GO terms, while columns represent the different biological processes associated with them.
